# Athlete maltreatment in sport: Protocol for a scoping review

**DOI:** 10.1371/journal.pone.0338616

**Published:** 2025-12-18

**Authors:** David M. Brown, Amy E. Nesbitt, Sasha Gollish, Tara-Leigh F. McHugh, Catherine M. Sabiston

**Affiliations:** 1 Faculty of Kinesiology and Physical Education, University of Toronto, Toronto, Ontario, Canada; 2 Rehabilitation Sciences Institute, University of Toronto, Toronto, Ontario, Canada; 3 Faculty of Kinesiology, University of Calgary, Calgary, Alberta, Canada; Government Law College, INDIA

## Abstract

Research on maltreatment in sport demonstrates detrimental effects on athletes’ well-being, and recent news reports highlight the pervasiveness of this issue. However, inconsistencies in defining and operationalizing athlete maltreatment in sport have resulted in issues with conceptual clarity that limits current research, practice, and monitoring within and across sport sectors. This scoping review will synthesize the scientific literature on athlete maltreatment in sport and identify current knowledge gaps for research and practice. The review objectives are to: (1) map how maltreatment has been conceptualized and operationalized in the literature; (2) identify and describe the types of athlete maltreatment that have been investigated; and (3) explore current trends in research approaches and methods applied to the study of athlete maltreatment. An established six-stage scoping review methodology will be applied, as well as field-specific guidelines for community advisory group consultations. The protocol will be conducted over a one-year time frame and has been registered in advance (https://osf.io/r7ycp/overview). Relevant sources will be identified using a systematic search strategy across six electronic databases. Study screening procedures will occur in duplicate using pre-determined eligibility criteria. For inclusion, articles must address the concept of maltreatment among athletes (of any age, sport, or competition level), and contain original peer-reviewed research. Extracted data will be analyzed using qualitative content analysis and descriptive statistics. Preliminary results will be presented to community advisors (e.g., athletes, coaches, sport administrators, clinicians, policy writers, researchers) to contextualize findings and prioritize actionable recommendations to improve sport systems. Results will be published in a peer-reviewed journal and presented at academic conferences for sport leaders and researchers with the aim of informing measures, interventions, frameworks, and/or policies.

## Introduction

Sport participation has the potential to significantly improve psychosocial and physical health and well-being of athletes [1–3]. Recently, several sports organizations have faced increased scrutiny for the presence of athlete maltreatment within their respective sports [4–10]. Athlete maltreatment encompasses relational (e.g., neglect, psychological, sexual, and physical abuse) and non-relational (e.g., harassment, bullying, discrimination) forms, which largely depend on the relationship between the athlete and the transgressor [11–12]. Of particular concern is the significant threat that maltreatment has on the psychological and physical health, safety, and overall well-being of athletes. For instance, athletes who have experienced maltreatment report self-harming behaviors [13], high levels of body dissatisfaction [14–18], disordered eating patterns (e.g., self-induced vomiting, laxative use) [15,17,18], and low self-worth [15] and self-esteem [16–19] as well as prolonged physical injuries from excessive exercise and training [18]. Athletes who experience maltreatment are at risk of developing psychological disorders, such as depression [15–16], eating disorders [14–16,18], and post-traumatic stress disorder [15]. In a survey of Canadian National Team Athletes, 75% of participants reported experiencing at least one form of maltreatment [10]. A comprehensive understanding of athlete maltreatment is essential for guiding future research and developing effective initiatives to safeguard sports.

Since the mid-1990’s there has been a surge of research on athlete maltreatment within the field of sport psychology, aligning with contemporary frameworks of maltreatment, and greater attention on national and international policy initiatives to prevent maltreatment [11,12,20–23]. While research has begun to elucidate the prevalence [10,17], impact [15,18], and consequences [14–16,18] of maltreatment in sport, several challenges and limitations remain [24]. First, existing studies have often focused mainly on relational maltreatment among specific groups of athletes (e.g., elite level athletes) [10,17,25]. This limits our understanding of the full scope of both relational and non-relational forms of athlete maltreatment across all sport levels and creates challenges for advancing policy and other safeguarding initiatives [25]. Second, as cited in a published narrative review [11] and a position statement [23], a wide range of theoretical frameworks, conceptualizations of maltreatment, and assessment tools have been employed in the study of maltreatment in sport; resulting in substantial variability and inconsistencies in how the concept of maltreatment is defined and operationalized both within and across studies. Third, while qualitative approaches provide unique insight into the experiences of athletes who have experienced maltreatment, advancements in quantitative investigations have been limited due, in part, to the current lack of validated measures of maltreatment appropriate for use within sport contexts [26]. Conceptual clarity and psychometrically validated questionnaires are required to capture the prevalence of maltreatment in sport and identify areas for intervention [26].

In sum, the study of maltreatment in sport is a critical area of focus with a potential to promote not only athlete safety but create thriving athlete environments that actualize sport benefits [11,27]. Currently, there is no consensus on the definition, application, and measurement of athlete maltreatment in sport. This is important to address as discrepancies in conceptualizations and measures of maltreatment pose a major limitation to current research, study comparisons, policy formation, and surveillance or monitoring systems within and across different sport types, organizations, competition levels, and countries. Despite the growing amount of research on the topic of maltreatment in sport, the literature remains fragmented, lacking a comprehensive synthesis to identify gaps, guide future research, and to help inform policy.

Given these limitations, a scoping review is necessary to systematically map the available evidence identifying various forms of maltreatment, affected populations, and contexts in which these behaviors have been investigated, and understand how the concept of athlete maltreatment has been studied within the literature. Specifically, a scoping review design offers a more comprehensive exploration of maltreatment within sports, elucidating various dimensions of this issue. Unlike traditional meta-analyses and systematic reviews, which often maintain a narrow focus, a scoping review allows for a broader exploration and synthesis of the extant literature [28]. Moreover, a scoping review may identify key constructs relevant to the development of more comprehensive measures/ assessment techniques to help standardize the process for identifying trends, testing naturally occurring experiments, and exploring the impacts of policies and programs over time [26,29].

### Study purpose and objectives

The overall purpose of this scoping review is to systematically map and synthesize the scientific literature on athlete maltreatment in sport, with the aim of identifying key themes, knowledge gaps, and directions for future research. Specifically, this review aims to: (1) map how maltreatment has been conceptualized and operationalized in the literature (e.g., definitions, theoretical frameworks); (2) identify and describe the types of athlete maltreatment that have been investigated; and (3) explore current trends in research approaches and methods applied to the study of athlete maltreatment (e.g., study designs, measures, athlete characteristics). Taken together, these research aims will help to inform measures, prevention and management interventions, frameworks for research and practice, and policy implications.

This scoping review is an important first step to understand how athlete maltreatment has been studied, with a particular focus on current trends and challenges in the conceptualization and operationalization of maltreatment. The findings from this review will provide valuable insights for improving the methodologies and practices in athlete maltreatment research, and are expected to inform future work focused on developing novel measurement strategies to enhance the accuracy of prevalence and outcome assessments.

## Materials and methods

### Study design

A scoping review is a type of knowledge synthesis, inclusive of broad study objectives and methodologies [30]. A scoping review design was selected due to the key strengths of this approach, namely (i) enhancing conceptual clarity, (ii) mapping conceptual and methodological trends in emerging areas of research, and (iii) identifying knowledge gaps and future research directions [29–31]. Further, a scoping review design has been recommended within sport psychology to summarize highly heterogeneous bodies of literature, as is seen in the study of athlete maltreatment in sport [29].

The scoping review protocol will follow standard methodological procedures, including six stages: (1) identifying the research question(s), (2) identifying relevant studies, (3) study selection, (4) charting the data, (5) collating, summarising and reporting the results, and (6) advisory group consultation [28,32]. Aligning with recent field-specific scoping review guidelines, this review will be conducted in consultation with an advisory group consisting of athletes, coaches, sport administrators, clinicians, policy writers, and researchers, prioritizing engagement and feedback from those who identify as survivors of maltreatment [29]. An iterative and reflexive approach will be applied throughout the review process in order to apply feedback from consultations to maximize the relevance and applicability of the results [33]. The Preferred Reporting Items for Systematic Reviews and Meta-Analyses – Extension for Scoping Reviews (PRISMA-ScR) outlines the best practices for conducting and reporting scoping reviews and will also be applied to promote methodological rigour and transparency [34]. The PRISMA protocol checklist can be found in online supplemental S1 Table.

The current protocol has received ethics approval from the University of Toronto’s Health Sciences Research Ethics Board (REB#47217) and has been registered through Open Science Framework (https://osf.io/r7ycp/overview). The scoping review will be conducted over a one-year time frame, approximately November 2024 to November 2025. The first two stages have been completed, including the database search (run on November 20, 2024). Study screening procedures (stage 3) are in the advanced stages of completion. Based on this timeline, participant recruitment for stage 6 advisory group consultations will take place in October 2025, after article data extraction and analysis.

### Stage 1: Identifying the research question

This scoping review will synthesize the scientific literature on athlete maltreatment in sport and identify current knowledge gaps for research and practice. Three specific research questions will be addressed: (1) How has athlete maltreatment been conceptualized and operationalized (e.g., defined) in research within the context of sport?; (2) What types of maltreatment have been investigated among athletes and in what sport contexts and/or settings?; (3) How has athlete maltreatment been studied, including any trends in the research purpose, conduct, approaches and methods applied (e.g., study designs, athlete characteristics; measurements used)? To frame the review research questions, and inform the study selection process, the population, concept and context of interest have been clearly defined below [31].

#### Population.

While maltreatment can occur to anyone in the sport setting (including athletes, caregivers, coaches, referees, etc.), the current review will focus specifically on experiences of maltreatment among athlete populations. Current and former athletes with experience in any sport context, during any developmental period, may be included. This scoping review will not limit studies based on the participants’ age, gender, sport type, or competition level.

#### Concept.

Broadly, maltreatment refers to “volitional acts that result in or have the potential to result in physical injuries and/or psychological harm” [35]. Athlete maltreatment is a global construct that can be divided into two subdomains: relational and non-relational maltreatment [12]. Relational maltreatment is subdivided into sexual, emotional, and physical abuse, and neglect; non-relational maltreatment is subdivided into institutional maltreatment, child labour, harassment, and bullying. Interpersonal violence, hazing, and discrimination are additional terms frequently used within the athlete maltreatment literature, which are encapsulated within this definition and subdomains. For this study, forms of maltreatment perpetuated online (e.g., via social media) will also be included (e.g., online emotional bullying by teammates) [36]. See online supplemental S2 Table for specific definitions and examples of each type of maltreatment.

#### Context.

Definitions of sport vary considerably across contexts and cultures [37]. For this scoping review, we broadly define sport as any organized competitive or recreational form of physical activity, involving clear rules of play/participation and structured form of engagement, that facilitates the formation of social relationships of any kind and is aimed at improving the physical health and mental well-being of those involved [38]. This review will consider a broad array of sports which may be further contextualized by training and competition metrics (e.g., team-based vs. individual; aesthetic vs. non-aesthetic, recreational vs. elite) such that dance, cheerleading, martial arts, and bodybuilding, and other structured fitness competition fit the definition of inclusion. Studies focused on athlete maltreatment in any sport setting, and at any time along the sport journey, will be considered relevant for this review [11].

### Stage 2: Identifying relevant literature

#### Information source.

Six electronic databases in fields related to sport, psychology, and health will be used: (1) MEDLINE, (2) EMBASE, (3) PsycINFO via Ovid, (4) CINAHL, (5) Sport Discus via EBSCO, and (6) ProQuest Dissertations and Theses Global. Peer-reviewed articles containing original research (e.g., empirical studies), as well as theses/dissertations will be included. Relevant journals (e.g., Journal of Interpersonal Violence; Psychology of Sport and Exercise) and reference lists of included sources will be manually searched for relevant articles missed in the initial search to enhance the comprehensiveness of the search on athlete maltreatment in sport.

#### Search strategy.

A systematic search strategy has been developed and conducted in consultation with a health sciences librarian. Search terms were explored within subject headings, titles, abstracts, and keywords. Terms for the population, concept and context were combined using appropriate Boolean logic and operators (e.g., ‘and’, ‘or’, ‘not’). See online supplemental S3 Table for the MEDLINE search strategy and list of keywords. All databases were searched November 20, 2024 (search yield following removal of duplicates: 10,817).

Given the known challenges and inconsistencies in how athlete maltreatment in sport has been defined and operationalized as this research area has evolved over time, the search strategy was peer reviewed by topic experts who are not on the review team. This peer review step provides valuable feedback to improve the overall rigor, integrity, and appropriate translation of the search strategy across all databases (e.g., CADTH Peer Review Checklist for Search Strategies) [39]. The search strategy was also pilot-tested using a multi-step process to examine the sensitivity and precision of the search to ensure relevant sources were not missed before being translated to all databases.

### Stage 3: Study selection

All identified sources will be uploaded in Covidence and collaboratively screened by a multidisciplinary research team using predetermined eligibility criteria [28,32]. Following data de-duplication, at least two reviewers will screen articles in two stages: (1) titles and abstracts and (2) full-text review. Ten sample studies will be used as a calibration exercise and decisions (i.e., included, excluded, uncertain) will be compared between reviewers. The research team will meet to clarify the criteria as needed. This process will be used to pilot the inclusion/exclusion criteria. Interrater reliability will be assessed and a score of 80% consistency will be required before formal title and abstract screening takes place. At least two reviewers will independently screen the full-text articles. The same calibration exercise will also be implemented at this stage and clarification meetings will occur as needed. Disagreements will be resolved by consensus between the reviewers or the decision of a third reviewer.

#### Eligibility criteria.

For inclusion in this scoping review, sources must address the concept of maltreatment among athletes within any sport context. Peer-reviewed research written in English that is published between 1993 and 2024 will be included. Language restrictions are necessary due to time and resource constraints. In the case that a full-text/English language copy cannot be found, the corresponding author will be contacted via email to obtain the article. For the purpose of the present scoping review, the date range has also been limited to reflect the time period where there has been a significant rise in athlete maltreatment research. Particularly, increased research attention to athlete maltreatment first emerged following several high-profile cases of athlete abuse in the 1990s, which was followed by several conceptual papers that gave rise to much of the research landscape today [11,20,26,40]. This data restriction will ensure that the review is broad enough in scope to capture how the study of athlete maltreatment has evolved over the past 30 years, while also synthesizing current evidence that can inform present-day measures, intervention, programs, frameworks, and/or policies.

Specific inclusion/exclusion criteria are detailed in Table 1 and Table 2. Throughout the review process, it is expected that the draft of the eligibility criteria will undergo slight revisions. Any modifications will be transparently reported in the final published review.

**Table 1:** Eligibility for Stage 1 Title/Abstract Review.

Inclusion Criteria	Exclusion Criteria
a) Population: Refers to current or former athletes of any age, sex, gender, competition level, or sport type. b) Concept: Athlete maltreatment is identified as a key focus within the purpose/objectives/research question, outcome measures, and/or findings. This includes relational, non-relational, and/or online maltreatment. c) Context: Is set in any sport setting. d) Type of source: Papers containing original research (e.g., quantitative, qualitative, or mixed method empirical studies), including peer-reviewed articles as well as theses/ dissertations. e) Publication language/date: Written in English and published between 1993 and 2024.	a) Population: Refers to non-athlete populations within sport (e.g., coaches, parents/caregivers, administrators, etc.). b) Concept: Athlete maltreatment is not a clear and explicit focus of the study. c) Context: Is not set in a sport setting. d) Type of source: Study protocols, review articles, commentaries, opinions, position statements, clinical guidelines, books/book chapters, grey literature, conference proceedings, articles solely focused on the development/psychometric testing of measures or assessments. e) Publication language/date: Written in a language other than English and was published prior to January 1^st^, 1993.

**Table 2:** Eligibility for Stage 2 Full-Text Review.

Inclusion Criteria	Exclusion Criteria
a) Population: Refers to a clearly defined athlete population with current or former sport participation, of any age, gender, competition level or sport type. b) Concept: Athlete maltreatment (including relational, non-relational, and/or other form) is clearly defined, operationalized, measured, and/or discussed as a key concept. c) Context: Is set in any sport setting.	a) Population: Refers to mixed/convenience samples that encompass non-athlete populations (e.g., coaches, parents/caregivers, administrators, students, general community samples) without stratifying results for interpretation. b) Concept: Broadly focused on similar constructs (e.g., childhood maltreatment, adverse childhood experiences) or related issues in sport (e.g., injury, athlete mental health). c) Context: Sport context is not clearly stated or emphasized.

### Stage 4: Data extraction

In accordance with recommended data charting methods, a standardised and systematic charting form will be used to organise, display, and interpret specific details from the included studies [28,32]. Data extraction will be completed by at least two reviewers using Excel. The charting form will be piloted using a small sample of included sources (i.e., 5–10 articles) to ensure consistency among reviewers. A draft charting form is shown in Table 3. Each section of the data charting form directly aligns with the three review objectives and is central to the analyses described in stage 5 of this protocol. Any challenges or disagreements during the data extraction process will be discussed with the research team until a unanimous solution is reached.

**Table 3:** Draft charting form.

General document details	
APA citation	Full author, date and journal details.
Country	Country of publication.
Academic discipline	Broad field of research or practice (e.g., kinesiology, psychology, sociology), based on the author affiliations and publication journal.
Conceptualization and operationalization of maltreatment in sport	
Conceptualization	How was maltreatment described within the context of sport?
Definition of maltreatment	Definition or operationalization of athlete maltreatment.
Theoretical framework/model	Any theories, conceptual model(s) or framework(s) applied.
Seminal papers referenced	Seminal conceptual papers that have informed the research (if applicable).
Research approaches and methods applied	
Purpose	The study purpose, research question(s), aim(s), and/or objective(s).
Population	Geographical location, sport and competition level, relevant demographic characteristics (e.g., age, sex, gender, racial and ethnic background, disability), and number of participants.
Study design	Quantitative, qualitative, or mixed methods.
Specific methodological approaches	Brief description of main experimental, intervention, observational or qualitative methods used.
	Intervention (if applicable): Description of key characteristics (e.g., intervention purpose/target, type, main components, duration)
	Intersectional approaches (if applicable): Description of how equity, diversity, inclusion, and accessibility were addressed. Specific methods (e.g., sampling, SGBA+) and/or theoretical frameworks applied to address diversity and identity factors among participants.
	Collaborative and participatory research approaches: Does the study involve people with lived experience of athlete maltreatment through consultation or partnerships? Description of any collaborative or participatory methods applied engaging athletes (or other knowledge users) as advisors or partners to draw on their lived experience expertise during the research process.
Instruments used to measure maltreatment	Specific measures/surveys employed (if applicable).
Context	The setting of the research, if provided.
Types of athlete maltreatment investigated	
Type	Any specific types of athlete maltreatment identified in the article. Informed by Stirling’s framework (and recent iteration by Kavanagh et al.), athlete maltreatment includes relational and non-relational domains which may encompass the following: emotional/psychological abuse, sexual abuse, physical abuse, neglect, assault, bullying, harassment, exploitation, institutional maltreatment, child labor, and discrimination.^12,36^
How/ where did the maltreatment occur?	Perpetrated either in physical (in-person) or virtual (online, social media) setting?
Summary of outcomes/ results	
Main outcomes	Any outcomes that were measured or described. Description of the consequences of maltreatment (if applicable).
Key messages/ important results	Description of main findings, take home messages, notable strengths and limitations, and implications (e.g., theoretical, methodological, practical).

### Stage 5: Collating, summarizing, and reporting the results

The final report will adhere to the PRISMA-ScR checklist [34], including a flow diagram detailing the explicit decision-making process involved in study selection. Data from the included articles will be analyzed using qualitative content analysis and descriptive statistics (e.g., counts, frequencies) [41,42]. Findings will be written as a narrative summary, with the inclusion of tables and figures. The final presentation of results will be determined in consultation with community advisory groups, and will likely involve a combination of written summaries, tables, and figures. Findings will be synthesized and discussed with respect to the three research questions outlined, along with considerations for future research, education, policy initiatives, and application/utility of the results to inform improved measurement of athlete maltreatment.

This scoping review is informed by a critical realist perspective, acknowledging that athlete maltreatment exists as a real and harmful phenomenon, while recognizing that its representation in research is shaped by social, institutional, and cultural contexts. We employ a reflexive and interpretive approach to data charting and synthesis. Rigor will be achieved through collaborative coding, iterative analysis, and critical dialogue within the research team to enhance credibility and dependability [43,44].

A single researcher will serve as the primary coder for this scoping review, consistent with a critical realist approach that emphasizes interpretive synthesis over quantitative reliability. Coding will be conducted reflexively, with analytic memos documenting the rationale for code assignment and the development of categories. The coding framework and emerging themes will be regularly discussed with the research team to enhance credibility and allow critical dialogue. An audit trail of decisions and coding processes will be maintained to ensure transparency, dependability, and trustworthiness of the findings [43,44].

NVivo software will be used to store and organize the data. The analysis will start with a familiarization process involving the reading and re-reading of the included articles. Next, initial codes will be developed inductively by reading and highlighting the article’s key information from the data charting form. Codes will be combined or split to create more descriptive and representative codes, and then organized into categories based on relationships and linkages between them. To address objectives 1 and 3, the open-coding and abstraction process involves primarily inductive analyses of the definitions, measures, and methods applied to the study of athlete maltreatment in sport (detailed in the data charting form). To address objective 2, types of athlete maltreatment identified in the charting form will be analyzed through the same process of open-coding. Analyses will be deductively informed by Stirling’s framework – allowing for the grouping of codes into relational and non-relational types of maltreatment, but also iteratively inductive for the emergence of new categories and subcategories as reported in the extracted literature [12]. In this way, a specific framework will not be confirmed or explored, rather used as a sensitizing framework to orient our thinking towards identifying distinct established and emergent forms of athlete maltreatment in the literature. Overall, this analytic approach can be especially useful in identifying current gaps and trends in how a complex and transdisciplinary concept, such as athlete maltreatment in sport, has been conceptualized and operationalized in research, and how understandings have evolved over time [29,45].

Lastly, this scoping review will include a quality appraisal of included studies using the mixed methods appraisal tool (MMAT) [46]. Consistent with the purpose of a scoping review, the results of the MMAT will not be used to exclude studies, stratify findings, or prioritize the interpretation of the results. The purpose of the appraisal is solely to provide a descriptive overview of the quality of literature on athlete maltreatment. The MMAT includes specific criteria for qualitative, quantitative, and mixed-methods studies and a table format will be used to display the appraisals. Importantly, no studies will be excluded due to low quality. This step will provide important insights into any strengths and challenges in the quality of current athlete maltreatment research [29].

### Stage 6: Community advisory group consultations

The multidisciplinary review team has been purposefully formed to ensure that the scoping review protocol has been developed collaboratively among researchers with content and methodological expertise in sport psychology, mental health and rehabilitation, and participatory research approaches/co-production. Importantly, each member of the review team also brings lived experience expertise from engaging in a variety of sport contexts and roles, from the perspective of an elite athlete, clinician, coach, referee, and sport/exercise psychology researcher.

Following Levac et al.’s [32] recommendations and recent field-specific guidelines proposed by Sabiston et al. [29], this scoping review will also involve consultation with community advisory groups (including athletes, coaches, sport administrators, clinicians, policy writers, and researchers) to integrate the lived experience expertise of end-users. Community advisory group consultations will be used to: (1) validate and present the findings of the scoping review, (2) highlight additional knowledge gaps and research priorities not identified by the review team, (3) inform how results can be applied to useful outcomes addressing athlete maltreatment, in sport, such as measures, programs, interventions, and/or policies, and (4) identify knowledge translation and dissemination efforts needed to reach diverse and broad groups of individuals in sport. While the advisory groups’ input will not influence coding procedures in stage 5, the feedback will be analyzed and integrated into the final report’s interpretation and presentation of the findings to ensure greater end-user representation. This consultation stage of the review process is recommended to enhance the relevance and applicability of the findings [47].

Consultative meetings will occur following stage 5 of the scoping review process (e.g., once preliminary analyses and syntheses have been carried out) using a qualitative focus group design with 18–30 community advisors involved in one of three planned discussions of 60–90 minutes in length [29,47,48]. To be eligible to participate, advisory group members must: (i) be aged 18 years old or older, (ii) be able to speak and understand English, and (iii) self-identify as a current/former athlete, or an expert/professional within the field of athlete welfare and maltreatment in sport (e.g., researcher, clinician, coach, sport administrator, policy writer). Participants will be recruited through the research team’s current networks, and using newsletters and social media advertisements.

Two focus groups will be conducted with experts/professionals and one separate focus group will be conducted with current and former athletes. A semi-structured focus group interview guide will be used to guide discussions broadly on (i) their overall impression of the findings, (ii) additional gaps in knowledge that were missed and/or future research priorities, (iii) how the results can be used for impactful outcomes such as questionnaires, frameworks, interventions and programs, and policy; and (iv) how the data findings should be shared. All focus groups will be audio and video recorded to produce a verbatim written transcript of the discussions. All data will be de-identified to protect participants’ identity before the analysis. NVivo software will be used to store and organize the data. Focus group transcripts will be analyzed using qualitative content analysis [42,43]. The final report will include a detailed description of the community advisory group consultation methods and results [29,32].

Given the sensitive nature of the topic, several protocols will be implemented to help manage potential emotional distress among participants. The consent form provides clear information about the material being discussed so potential participants can choose whether to participate in the study or prepare themselves for the upcoming discussion. Prior to the focus group discussion, participants will be reminded of their right to skip any questions asked of them, take a break from the focus group, or withdraw from the study at any time without penalty. Last, during the focus groups, a second researcher will check-in with participants to ensure emotional well-being in maintained, monitor the discussions and participants’ reactions for signs of distress, intervening if necessary by inviting participants to breakout rooms for independent consultation, taking notes, and maintaining a respectful environment protective of all participants’ well-being.

The review team will engage in ongoing reflexive practice to address the potential impact of our own positionality in shaping study decisions, consultations, and the interpretation of results [49,50]. Reflexivity will support the methodological rigor of this scoping review by challenging possible biases/assumptions on the topic of athlete maltreatment, and critically examining where positions of power and privilege influence research activities and how this can be addressed. The review team will also apply specific recommendations for enhancing the trustworthiness of qualitative content analysis and keep an audit trail detailing decision-making processes [43,44,51].

### Patient and public involvement

The scoping review design and protocol development has not involved patients or members of the public outside of the review team. As part of stage 6 of this protocol, current/former athletes (who may have witnessed or experienced maltreatment in sport), as well as other experts/professionals (coaches, clinicians, researchers, policy writers, administrators), will be engaged as community advisors. Their perspectives and feedback will shape the final interpretation and dissemination of review results, ensure that findings are relevant and applicable to the next stage of this research (focused on advancing the measurement of athlete maltreatment in sport), and identify future research priorities for protecting athlete welfare.

### Ethics and dissemination

The Health Sciences Research Ethics Board at the University of Toronto approved stage 6 of this scoping review protocol (REB#47217), which involves the collection of primary data from community advisory groups. Participants will provide written informed consent and complete a brief demographic questionnaire using a secure online survey (administered through University of Toronto Research Electronic Data Capture (REDCap) platform) before joining a consultative meeting. Verbal agreement with the study purpose and procedures will also be obtained in the focus groups prior to starting the discussion. Results of the review will be disseminated through traditional approaches, including open-access peer-reviewed publication(s) in academic journals, presentations at national/international conferences, and via a plain-language summary report presented on the researchers’ websites (e.g., infographics). Additional knowledge translation strategies will be developed based on community advisory group feedback and will aim to prioritize sharing review findings with coaches, athletes, and sport community members.

## Discussion and conclusions

The overall purpose of this scoping review is to systematically map and synthesize the scientific literature on athlete maltreatment, with the aim of identifying key themes, knowledge gaps, and directions for future research. These research aims will help to inform measures, prevention and management interventions, frameworks for research and practice, and policy implications. Despite these important impacts, the current review anticipates several limitations worth discussing. First, some behaviors that could constitute maltreatment are normalized within sport culture, such as excessive exercise, restrictive dieting, and public weigh-ins (among others) [18]. If such harmful behaviors are entrenched within specific sport contexts, they may not be explicitly self-reported or recognized as maltreatment by participants and/or researchers. Consequently, inconsistencies in the terminology used to characterize maltreatment in sport may contribute to articles being missed by systematic search strategies and lead to underrepresentation of studies. Second, while quality appraisal of the extracted articles will be conducted to help describe limitations to the field, none of the articles will be excluded due to low quality [32]. As such, the interpretation of the findings may need to be tempered but will be guided by the community advisory group consultations.

These limitations aside, there are strengths of this scoping review. By encompassing a diverse range of study designs and methods (i.e., qualitative, quantitative, and mixed-methods), this scoping review will provide an inclusive summary of the research landscape focused on athlete maltreatment. The review will directly address the lack of conceptual clarity and definitional inconsistencies in the field by mapping how maltreatment has been defined, measured, and studied. Further, by synthesizing operational definitions used across the literature, the findings could provide important foundational information to develop a standardized measure of athlete maltreatment. For researchers, the findings will stimulate novel questions for further inquiry and will highlight potential strategies to develop and test appropriate interventions and prevention frameworks. For sport organizations, these findings can inform the development, implementation, and evaluation of training programs for relevant leaders in sport, such as coaches, parents, officials/referees, and athletes. Finally, for policy developers, these findings can help underscore the need for stronger safeguarding policies to protect athletes across sport contexts. Consultation with the advisory groups will help ensure the relevance and applicability of these contributions [29,52].

Ultimately, this protocol outlines a rigorous and transparent process for conducting a scoping review on athlete maltreatment, generating insights that may guide future research, interventions, prevention strategies, and policy development.

## Supporting information

S1 Table PRISMA-P checklist. (PDF)

S2 Table Defining the scope of the review: population, concept and context. (PDF)

S3 Table MEDLINE (Ovid) database search strategy. (PDF)
