# Supplementary material for: Athlete maltreatment in sport: Protocol for a scoping review
Source: PLoS One. 2025 Dec 18;20(12):e0338616. doi: 10.1371/journal.pone.0338616 (PMC12714210; doi:10.1371/journal.pone.0338616)
Supplement: S2 Table — (PDF) [file pone.0338616.s002.pdf]

Table S2

Defining the scope of the review: population, concept and context.

**PCC Component**	**Definition**
**Population: Athletes**	Athletes of any age, gender, sport type, or competition level.
**Concept: Maltreatment**<br><br><br><br><br>**Relational**<br><br><br><br><br><br><br><br><br><br><br><br><br><br><br><br><br><br><br><br><br><br><br><br><br><br><br><br><br>**Non-Relational**	Maltreatment is defined as "volitional acts that result in or have the potential to result in physical injuries and/or psychological harm" (Crooks & Wolfe, 2007, p. 640).<br>Informed by Stirling's (2009) conceptualization, athlete maltreatment may refer to several specific forms:<br>- ***Sexual abuse*** is defined as any sexual interaction with person(s) of any age that is perpetrated against the victim's will, without consent or in an aggressive, exploitive, manipulative, or threatening manner.<br>- ***Emotional abuse*** is defined as a pattern of deliberate non-contact behaviours (e.g., berating, disparaging) within a critical relationship that has the potential to be harmful, regardless of intention of the perpetration to inflict harm and of evidence of the eventual harm inflicted. Emotionally abusive behaviour is categories into verbal emotional abuse (e.g., belittling, degrading comments, verbal humiliation) and non-verbal emotional abuse (e.g., physical emotional abuse and denial of attention and support).<br>- ***Physical abuse*** is defined as contact (e.g., striking, slapping, spanking) and non-contact (e.g., denying athletes access to water, food, or sleep; forcing athletes to train extra or at a higher intensity as punishment or frustration/anger) behaviours that inflicts physical harm or injury, discomfort, or trauma.<br>- ***Neglect*** is defined as passive or passive/aggressive inattention or omission of care to individual's needs, nurturing, or well-being that results in significant harm or risk of harm. Neglect can be subdivided into physical (e.g., delaying medical assessment/treatment for a sport injury), educational (e.g., encouraging or forcing athletes to miss education/classes for training), emotional (e.g., chronic lack of attention), or social neglect (e.g., allowing athlete to disregard sport results, regulations, and standards).<br>- ***Institutional maltreatment*** is defined as the abusive or neglectful experience of a person by serving institutions, when there is a failure to meet appropriate standards of care, or when the core practice of an organisation is abusive (e.g., competitions occur in unsafe facilities, failure of an organisation or report and address instances of athlete maltreatment).<br>- ***Child labour*** is defined as children who work for long hours, in dangerous or unhealthy conditions, or in an environment

	where they are exposed to lasting physical or psychological harm and can be divided into bonded (e.g., forcing child athletes to work against debt taken by their family) or non-bonded (e.g., financial rewards for child athletes for training or competing in hazardous conditions) child labour.<br>- ***Harassment*** is defined as any comment, conduct, or gesture directed towards an individual or group of individuals, which is insulting, intimidating, humiliating, malicious, or degrading or offensive. It can be subdivided into physical (e.g., pushing, threatening), sexual (e.g., lewd comments, inappropriate physical touching or fondling, repeated suggestion to engage in sexual conduct), emotional (e.g., telling embarrassing stories about athletes, put-down jokes), gender (e.g., exclusion based on gender), racial (e.g., referring to someone's race in a negative, vulgar, or derogatory terms), homophobia (e.g., exclusion based on sexual orientation).<br>- ***Bullying*** is defined as repeated physical, verbal, or psychological attacks or intimidations, without provocation by the victim, that are intended to cause fear, distress, or harm to the victim. Bullying can be categorised as physical (e.g., theft, hitting), emotional (e.g., teasing, threatening), or social (e.g., hazing/initiation rituals).
**Context: Sport**	Sport is defined any organized competitive or recreational form of physical activity, involving clear rules of play/participation and structured form of engagement, that facilitates the formation of social relationships of any kind and is aimed at improving the physical health and mental well-being of those involved (Lagaert & Roose, 2016). This review will consider any sport context as relevant.
