# Supplementary material for: Athlete maltreatment in sport: Protocol for a scoping review
Source: PLoS One. 2025 Dec 18;20(12):e0338616. doi: 10.1371/journal.pone.0338616 (PMC12714210; doi:10.1371/journal.pone.0338616)
Supplement: S3 Table — (PDF) [file pone.0338616.s003.pdf]

Table S3

MEDLINE (Ovid) database search strategy

#	PCC conceptual term of interest	Search term entered into OVID-Medline
**1**	Concept (maltreatment)	Harassment, Non-Sexual/ or Sexual Harassment/ or Rape/ or Sex Offenses/ or Physical Abuse/ or Violence/ or Gender-Based Violence/ or Workplace Violence/ or Bullying/ or Cyberbullying/
**2**	Concept (maltreatment)	((mental* or physical or verbal or emotional or sexual or psychological or material) adj2 (harm or abus*)).tw,kf.
**3**	Concept (maltreatment)	(assault* or maltreat* or mistreat* or ill-treat* or harass* or rape or sexual misconduct or violence or bully* or exploitation or neglect or coerc* or extort* or "child labour" or "child labor" or stigmati* or ostraci* or hazing).tw,kf
**4**	Concept (maltreatment)	(discriminat* adj3 (gender or sex or sexual or race or racial or weight or athlete)).tw,kf.
**5**	Population (athletes)	exp Athletes/ or exp Para-Athletes/
**6**	Population (athletes)	(athlete* or para-athlete*).tw,kf.
**7**	Context (sports)	Sports/ or Recreation/ or Athletic Performance/ or Sports for Persons with Disabilities/ or Youth Sports/ or Team Sports/ or Gymnastics/ or Baseball/ or Basketball/ or Boxing/ or Cricket Sport/ or Football/ or Golf/ or Hockey/ or Martial Arts/ or Mountaineering/ or exp Racquet Sports/ or Rugby/ or Skating/ or exp Soccer/ or exp Snow Sports/ or "Track and Field"/ or Volleyball/ or Diving/ or Water Sports/ or Weight Lifting/ or Wrestling/
**8**	Context (sports)	(sport* or parasport* or para-sport* or physical recreation or athletics or athletic performance or archery or badminton or baseball or basketball or bobsleigh or boxing or bowling or caving or cheerleading or cricket or dance or dancing or diving or fencing or football or golf or gymnastics or handball or hockey or horseback riding or judo or karate or kickboxing or lacrosse or martial arts or mountaineering or rock climbing or sport climbing or rowing or rugby or skateboard* or skating or ski or skiing or snowboarding or soccer or softball or squash or tennis or "track and field" or triathlon or volleyball or weight lifting or wrestling).tw,kf.

**9**	Context (sports)	((running or runner or cycling or cyclist or swimming or swimmer) adj2 (professional or olympic or competitive or elite or recreational)).tw,kf.
**10**	Concept	1 or 2 or 3 or 4
**11**	Population and Context	5 or 6 or 7 or 8 or 9
**12**	Combine PCC	10 and 11
**13**	Limit by language and date	Limit 12 to (english language and yr="1993-current")
